# Parental perceptions and experiences of an oral health care promotion intervention for children with congenital heart defects

**DOI:** 10.1080/17482631.2022.2070968

**Published:** 2022-05-13

**Authors:** Essi Karikoski, Kristiina Junttila, Mirkka Järvinen, Taisto Sarkola, My Blomqvist

**Affiliations:** a Children’s Hospital, Department of Children and Adolescents, Helsinki University and Helsinki University Hospital, Finland; b Nursing Research Center, Helsinki University and Helsinki University Hospital, Finland; c The Finnish Federation of Oral Health Care Professionals, Helsinki, Finland; d Minerva Foundation Institute for Medical Research, Helsinki, Finland; e Children’s Hospital, Department of Oral and Maxillofacial Diseases, Helsinki University and Helsinki University Hospital, Helsinki, Finland

**Keywords:** Health promotion, parents, qualitative research, paediatric dentistry, congenital heart defects

## Abstract

**Purpose:**

Congenital heart disease (CHD) is one of the most common congenital anomalies in children. Children with major CHD are at risk for developing endocarditis. Acute endocarditis may be life threatening and lead to heart failure. The purpose of this study was to explore parental perceptions and experiences of an early oral health promotion intervention (OHPI) targeting children with major CHD at risk for developing endocarditis later in life, and use this information to examine intervention feasibility.

**Methods:**

Nine parents (three fathers and six mothers) participating in a one and a half year OHPI were purposefully selected for qualitative evaluation of intervention feasibility using semi-structured interviews. The interviews were analysed with an inductive content analysis method.

**Results:**

The analysis resulted in four main categories and 14 subcategories that describe parental perceptions and experiences of the OHPI. The four main categories were timing of first intervention contact, effortlessness of intervention process, individuality of support, and relevancy of support.

**Conclusion:**

Parents of children with CHD perceived the OHPI as important and feasible to be implemented in daily life in children with systemic diseases overall. Further studies on timing of first contact and use of additional Web-based support are needed.

## Introduction

Congenital heart disease (CHD) is one of the most common congenital anomalies in children, with an incidence of 7–8 major cases per 1000 live births (Van der Linde et al., 2011). Children with major CHD are at risk for developing endocarditis. Acute endocarditis may be life threatening and lead to heart failure. According to the American Heart Association’s guidelines for endocarditis prophylaxis, the risk for bacteraemia is more common during daily oral hygiene routines, such as tooth brushing, than during dental procedures (Lockhart et al., 2008; Wilson et al., 2007).

A systematic review on dental caries prevalence indicated higher caries prevalence in children with CHD compared with healthy children. Furthermore, children with operated complex CHD seem to experience even more caries (Karikoski et al., 2021).

Children with CHD are potentially challenged with several risk factors for dental caries development during early years of life including frequent dietary intake (Evans et al., 2013; Hansson et al., 2012; Schulz-Weidner et al., 2020), frequent use of medications (Nederfors, 2000), and developmental tooth enamel defects (Hallett et al., 1992). Furthermore, high CHD disease burden may directly or indirectly increase the risk of child oral health care neglect in the form of inadequate caries prevention routines in the family.

Dental caries may lead to pain, oral dysfunction including difficulties to eat, and social impairment. Poor oral health is associated with dental fear and decreased quality of life (DaFonesca et al., 2009). Longitudinal studies in healthy children show that caries experience in primary dentition increases the risk of caries in the permanent dentition (Lin et al., 2021; Skeie et al., 2006). Caries during early life may involve procedures performed under general anaesthesia due to difficulties in co-operation related to age and development (Phantumvanit et al., 2017; Savanheimo & Vehkalahti, 2014). Maintaining a good oral health could also prevent hospitalization and dental procedure related anxiety. With early effective caries prevention, these risk dental procedures for children with CHD could potentially be avoided.

Health promotion is defined as a process of enabling people to improve and increase control of health problems. The goal of oral health promotion is to strengthen knowledge in the field and to motivate individuals to adapt favourable oral health behaviours that contribute to improved long-term oral health (Petersen, 2008). Multistage oral health promotion interventions have been reported to improve child oral health behaviour (Davies et al., 2005; Ghaffari et al., 2018; Plonka et al., 2013). A study from Australia showed that six-monthly health promotion interventions provided during home visits or by telephone contacts from birth to 18 months of age in addition to providing toothbrush and toothpaste prevented caries at two years of age (Plonka et al., 2013).

In an observational controlled study including 5–16-year-old children with CHD, Suvarna et al. (2011) showed that poor oral health was more prevalent among CHD children indicating a lack of sound knowledge for maintaining oral hygiene. The study also reported improved oral health following preventive treatments. One study in children with operated CHD evaluated the effectiveness of a standardized preventive oral hygiene program including dental care and showed that instructions for tooth brushing and motivation influenced plaque values and inflammation of the gingiva (Schultz-Weidner et al., 2021). Another study in children with CHD observed improved oral hygiene, reduced gingival bleeding and less untreated dentine caries following early participation in an oral health program compared to controls. However, the study was unable to show benefits for the early prevention of dental erosion or caries (Sivertsen et al., 2018).

Despite evidence of effectiveness of oral health interventions, little is known about caregivers’ experiences and perceptions of oral health promotion interventions targeting their children. The motivational interviewing (MI) technique includes the creation of a confidential relationship between the client and the counsellor. In the process, client self-efficacy is improved by counsellor mediated empathy and support. The overall goal is to increase patient intrinsic motivation. The client is guided to identify personal motivation for change with information provided to achieve such change. Open ended questions, reflective listening, affirming and reiterating statements are provided in order to increase client motivation to adapt favourable habits (Miller & Rollnick, 2013).

In a study exploring parental perceptions of MI intervention for obesity prevention among 2-6-year-old children, 66% of the parents perceived the visits very helpful in reducing child sugar-sweetened beverage intake. More than half of the parents reported intervention satisfaction and more than 90% recommended the intervention to peers (Woo Baidal et al., 2013).

The ORALPEDHEART-study is a multistage oral health promotion intervention (OHPI) study that includes a combination of face-to-face and phone call oral health promotion counselling using the MI technique. The randomized controlled trial combines experts from paediatric dentistry, health sciences and medicine to promote oral health in children with complex CHD as well as in children with any operated CHD combined with a syndrome, both considered at risk for endocarditis and dental caries. In addition to repeat personal parental counselling, written information and home delivered toothbrushes and toothpastes were included in the intervention.

A deeper understanding of OHPI feasibility and applicability in the daily life of children with CHD could provide valuable information. The qualitative exploration and evaluation of parental perceptions and experiences combined with feedback analysis could provide insight and cues for improvement and further development of the intervention. Thus, the purpose of this study was to explore parental perceptions and experiences of OHPI and use this information to examine the feasibility of the OHPI among families with CHD children at highest risk for endocarditis. The research questions were: “What perceptions and experiences about the OHPI do the parents have?” and “What are parents’ suggestions for improvement of the OHPI to make it more feasible and applicable?”

## Material and methods

### ORALPEDHEART-study

This qualitative study was conducted in the Children’s Hospital, Helsinki University Hospital, Finland as part of the ORALPEDHEART-study (Clin Gov Trial NCT03329170). Children born in Finland with a) major CHD and potentially included in the criteria of endocarditis prophylaxis (Wilson et al., 2007), or b) with any operated CHD combined with a syndrome, were offered the opportunity to participate in the study within 12 months from birth. Aims, inclusion and exclusion criteria of the study are presented in Table I. Demographic descriptive data was prospectively collected in the OHPI at baseline. Parents in the same household were instructed to complete the data collection questionnaires if they were both present at the time of the recruitment. Child health information was collected from hospital charts.

**Table I.** Aims, inclusion and exclusion criteria of the ORALPEHDEART-study

**Aims** The ORALPEDHEART-study is a randomized controlled intervention trial with the primary aims to explore the effect of six-monthly counselling and support to improve awareness and maintenance of good oral health, prevent the development of poor oral health including dental caries, decrease the need for operative dental care, prevent dental anxiety, and improve oral health related quality of life. The secondary aim is to elucidate factors associated with the development of poor oral health and/or orofacial dysfunction as well as family attitudes and needs of support. The intervention is offered during early age to families with a child with complex CHD, or any operated CHD combined with a syndrome.	
**Inclusion criteria** Age < 12 months and prosthetic cardiac valve or patient likely to undergo valve surgery using foreign material including homograft (potentially included in the criteria of endocarditis prophylaxis) Age < 12 months and unrepaired cyanotic CHD including palliative shunts and conduits Age < 12 months and repaired CHD with residual defects at the site or adjacent to the site of the prosthetic patch or device which inhibits endothelialization Age < 12 months and cardiac transplantation recipient or listed for transplantation (potentially included in the criteria of endocarditis prophylaxis) Age < 12 months and cardiomyopathy (potential cardiac transplantation recipient) Age < 12 months and chromosomal abnormality or syndrome and any invasive intervention (surgery or cath) for CHD or likely to require invasive intervention for CHD	**Exclusion criteria** Neither parent able to comprehend intervention instructions provided in Finnish Child in out-of-home care (e.g., foster care)

### Intervention description

**Figure 1.**
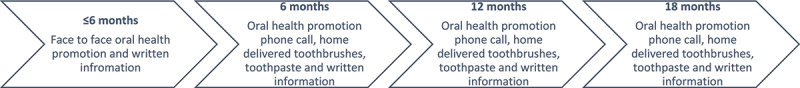
The gradual progress of the one and the half year dental hygienist led oral health promotion intervention.

The intervention group received a one-and-a-half-year dental hygienist led OHPI in four stages including one face-to-face oral health promotion session at baseline followed by three oral health promotion phone calls at six, 12 and 18 months from birth. The gradual progress of the OHPI is presented in Figure 1. The researcher (EK), a registered dental hygienist as a background, performed the recruitment of the participants for the ORALPEDHEART-study and the OHPI. EK has completed additional training on the method (motivational interviewing) of oral health promotion used in the OHPI.

At baseline, families randomized to the intervention group were during the in-hospital recovery phase of the primary cardiac surgical. Intervention offered verbal and comprehensive written information on the importance of oral health in children with CHD. Oral health promotion themes included caries development, role of eating habits in caries prevention, and oral health care.

At the age 6, 12 and 18 months, parents in the intervention group were sent a letter containing toothbrushes, toothpaste, and the oral health information pamphlet provided also at baseline. Parental phone counselling was then provided by dental hygienist (EK) using the MI technique including the themes mentioned above. The aim was to improve parental knowledge of oral health maintenance in children with CHD. In addition, at 6 months the child´s local primary health care clinic was contacted to inform about the participation in the study. These clinics monitor the growth and development of children and discuss matters related with health overall, well-being, parenting in general, and recommendations on oral health.

### Qualitative study design and sample

A qualitative descriptive study design with semi-structured interviews and inductive content analysis was applied to gain an understanding of the parents’ perceptions and experiences of the OHPI. Fifteen families were selected by purposeful sampling among families randomized to the intervention group in the ORALPEDHEART-study after completing the intervention process (Elo et al., 2014; Elo & Kyngäs, 2008). All 15 families who had completed the one-and-a-half-year intervention process by November 2020 were offered the opportunity to participate in this interview study. The selection criteria were completion of intervention process and the that interviewed parent had received the oral health promotion from the dental hygienist during the intervention by telephone.

When the child turned 24 months of age, the caregiver was contacted by the researcher (EK) with a letter including information to participate in a recorded phone call interview. Nine families, including six mothers and three fathers, consented to participate. As recruitment was done by letter, the researcher did not have the opportunity to inquire the reason for participation decline.

### Ethics

The qualitative study was approved by the Research Ethics Board (HUS/96/2017) and the hospital (HUS/149/2017) as part of the ORALPEDHEART study. Participation in the study was voluntary and confirmed by the signing of an informed consent.

### Data collection

A semi-structured standard interview guide was developed by authors EK, KJ and MB. The interview included eight main questions. The questions were designed to provide answers to the research questions, and they arise from the purpose and the topic of the study. The corresponding author (EK) performed three pilot interviews with parents outside the study. The content of the interview guide was refined based on multiple author reviews of pilot data and the questions. All interviews were introduced with the same question with the order of the seven remaining questions individualized based on parental responses during the interview. Five main questions with cues to encourage more in-depth responses was designed to explore parental perceptions and experiences about the intervention. The five main questions were the following: 1) How do you feel about the early timing of the oral health counselling intervention? The purpose of the question was to explore parental perceptions and experiences about the early introduction of the intervention. 2) How do you feel about the 6, 12 and 18-month follow-up counselling contacts during the intervention process? 3) What thoughts and feelings have raised during the different counselling stages? 4) Describe your most negative experience of the intervention, and 5) Describe your most positive experience of the intervention. The purpose of the main questions 2–5 was to explore parental perceptions and experiences about the intervention process.

The remaining three main questions were designed to explore suggestions for improvement and feedback regarding the intervention. These main questions were the following: 1) Could you share your thoughts if the intervention model (OHPI) applied for CHD families could be successfully applied to families with other significant systemic child diseases? 2) Could you describe an alternative better way of providing counselling to improve oral health in children with CHD? 3) Would you recommend OHPI to families with other significant systemic child disease and why do you think these families would benefit or not benefit from OHPI?

Data regarding the oral health promotion MI intervention process was also collected during each oral health promotion phone call. This included documentation of duration of phone call in minutes and documentation of main parental concerns regarding their child’s oral health.

Qualitative interview data was collected by one of the authors (MJ) between October 2019 and November 2020 with semi-structured phone call interviews. Interviews were recorded and all interviews were conducted in parental native Finnish language. The duration of the interview was between 10 to 20 minutes. Data collection continued until the data was saturated and the answers started repeating themselves.

### Analysis

Interview audiotapes were transcript verbatim by the first author (EK). Inductive content analysis was used to identify parents’ perceptions and experiences of the OHPI. In inductive content analysis categories that describe the studied phenomena are identified and analysed from interview data. The identified categories are the results of the analysis. The data analysis process proceeds from specific to general in order to identify categories that are named to describe the phenomena in the most descriptive way (Elo & Kyngäs, 2008).

Authors EK, KJ and MB analysed the data from specific to general and continuously discussed the classification of the sub- and main categories (Elo et al., 2014; Elo & Kyngäs, 2008). First, all interviews were combined into one text including 46 pages (size A4 with 1.5 spacing) for all transcript responses combined. This text was read several times to create a general impression. Second, important sentences for the purpose of this study (parents’ perceptions and experiences about the intervention) were identified and highlighted in different colours in the combined document. Third, the highlighted original expressions were abstracted into more general descriptions. Fourth, highlighted original expressions and abstracted descriptions were combined and organized into subcategories. Fifth, subcategories were then named using content-characteristic expressions. In the final sixth phase, the subcategories were grouped as main categories and named descriptively (Elo & Kyngäs, 2008). An example of the analysis process is presented in Table II.

**Table II.** An example of the analysis process

Original expression	Abstracted description	Subcategory	Main category
*“The fact that it has been always reminded about it … about brushing of teeth” (Parent 7)*	Reminder of brushing teeth		
*“it is always a good reminder” (Parent 5)*	Good reminder		
*“The phone call is like … ok, now I need to remember those things” (Parent 1)*	After the phone call remember again		
*” It is always a good reminder of it … that everyone is noticed individually … ” (Parent 5)*	Good reminder that has been performed according to a personal situation	Maintaining awareness	Relevancy of the support
*”so … that there has been like regular reminder … that this oral health is important thing … even though we have a lot of different schedules in our daily life … so it is good and important … ” (Parent 2)*	Regular reminder about important matter in the middle of the hectic daily life		
*“I think that it has been good … that it has always been reminded of it … that this is the information pamphlet … and the phone calls … and these are the things I need to remember” (Parent 1)*	Good reminder		

## Results

Table III outlines the background information and representativeness of the parents that participated in this qualitative substudy within the ORALPEDHEART. If both parents answered the survey at baseline the data from the first respondent was used in descriptive statistics.

**Table III.** Background information and representativeness of parental qualitative interview sample

Background information	Interviewed parents N = 9	OPH participating parents N = 71
**Age** Under 30 y or 30 y 31–35 y 36 or over 36 y	2 3 4	26 18 27
**Education** Grammar school Vocational school Matriculation examination University of Applied Sciences or University	– 4 – 5	3 27 7 34
**Smoking** Yes No	1 8	9 62
**Family income per year** Less than 20 000€ 20 000–39 999 € 40 000–59 999 € 60 000–79 999 € 80 000–99 999 € Over 100 000€	– 3 2 4 – –	-16 32 15 6 2
**Child CHD complexity grade** 2 3 4	2 5 2	20 31 20
**Child age at primary heart procedure** 0–1 months 2–3 months 4–6 months Over 7 months	4 3 2 –	38 11 15 7
**Days spent in the hospital in conjunction with primary heart procedure** ≤ 10 11–20 21-30 31–40 41-50 ≥51	2 2 1 2 1 1	16 21 10 11 3 10
**Days spent in the PICU in conjunction with primary heart procedure** ≤ 10 11–20 21-30 31–40 41-50 ≥51	5 3 1 – – –	49 10 7 1 1 3

CHD complexity grade 2 = simple CHD with complete surgical repair, grade 3 = complex CHD with complete surgical repair likely requiring reintervention, grade 4 = complex CHD without complete surgical repair (e.g., surgical palliation including single ventricle physiology), OPH = ORALPEDHEART, PICU = Paediatric intensive care unit

In all, four main categories and 14 subcategories emerged that describe the parents’ experiences of the intervention. The four main categories were timing of the first contact, effortlessness of the intervention process, individuality of support, and relevancy of support (Table IV).

**Table IV.** Description of the subcategories and main categories

Subcategory	Main category
Child’s appropriate age Many new things to think about Burden of time spent in hospital Blurriness of recollections	Timing of the first contact
Opportunity to be prepared for the counselling Easiness of phone counselling Interaction with another person Personal support Regularity	Effortlessness of the intervention process
Comprehensiveness Essentiality	Individuality of the support
Maintaining awareness Usefulness of the provided information Emphasis on the importance of oral health in everyday life	Relevancy of the support

### Timing of the first contact

The category “timing of the first contact” describes the parents’ experiences when they were offered the opportunity to participate in the study and at baseline presented with the verbal and written information regarding oral health of children with CHD. Parents were contacted during the child’s hospital recovery from primary cardiac surgery. The category included four subcategories: 1) child’s appropriate age, 2) many new things to think about, 3) burden of time spent in hospital, and 4) blurriness of recollections.

#### Child’s appropriate age

At the time of the first contact, the children were between two weeks to 10 months of age. The parents described that the age of the child and the timing of the OHPI’s first phase (children under 12 months of age) was appropriate at the time of the participation.

At least in terms of age it was appropriate, although there seemed to be a bit of a hurry and the situation was busy … However, it was quite good that this issue was addressed at an early stage (Parent 1)

#### Many new things to think about

The parents expressed that during the OHPI´s first contact, there were many new things to think about due to their child’s health condition. Parents also described that the time the family spent in the hospital was very busy and it was difficult to digest all the information provided.

It was a bit confusing situation and it was only a short time since the baby was born and we had been in the ward for a short time … everything was new to us anyway … with children with heart disease … It was like on a roller coaster ride ….an then we received a huge bunch of papers from different parties … and they were all provided within a short time period … it was like … WHAT?! (Parent 4)

#### Burden of time spent in hospital

The parents expressed that the time spent in hospital was somewhat exhausting. They said that they constantly encountered new people and that the unstable and variable situation of their child’s health condition was stressful. Parents also emphasized feelings of being in the middle of a crisis and the need to focus all energy to stay on track of things related to their child’s heart condition.

We were no longer in shock regarding the difficult situation of our child …. However, we were far from home in totally different environment … there was always a new person … a nurse or a doctor … always … and then there was constantly different kinds of procedures … or someone asking to participate for research or something … (Parent 7)

#### Blurriness of recollections

The parents reported that their hospital recollections were blurry and that they were having difficulties to recall all the events that took place at that time. Their recollections of the situation when they met the researcher for the first time and were given oral health care information were also blurry. Written information and participation in the study were the most memorable matters.

Well … I don´t really remember … I really don´t remember a lot from the days we spent in hospital … (Parent 1)

### Effortlessness of the intervention process

The category “Effortlessness of the intervention process” describes parental experiences of the intervention as an ongoing process. The category included five subcategories: 1) opportunity to be prepared for the counselling, 2) easiness of phone counselling, 3) interaction with another person, and 4) personal support and 5) regularity.

#### Opportunity to be prepared for the counselling

The parents were pleased that they were informed about the oral health promotion phone call in advance providing the opportunity to think about possible questions in advance.

… I’ve been home with a child, so answering the phone has been easy … I´ve been notified by email that a call will come shortly … when I knew that the phone call would be performed soon … I might have thought in advance … like problems and questions that we have considering oral health of our child … (Parent 2)

#### Easiness of phone counselling

The parents experienced it pleasant to handle issues regarding their child’s health over the phone instead of an additional scheduled hospital appointment. They expressed that the time for the phone call was easy to arrange because of the maternity leave. Parents also experienced that it was convenient to discuss with the oral health care professional during working hours.

The parents experienced that they had many things to figure out related to their child’s condition, so they appreciated the help and information offered. Furthermore, they said that the intervention process proceeded smoothly and effortlessly.

Yes … because we have a lot of special things and different appointments related to child´s heart condition … to take care of … and everything … so … what it would have been like … if we would have needed to go to some scheduled appointment … it would have been very difficult for us … this has been much easier to handle over the phone (Parent 6)

#### Interaction with another person

The parents said that it was nice to discuss oral health care issues with another person. They appreciated the opportunity to ask questions about oral health directly from a real-life person instead of using a web-based contact method (e.g., email or message on the answering machine or feedback portal). Regular contact from a real-life person was very important when it came to effortlessness of the intervention process.

It has been great that a real person has called … and I haven’t had to fill out forms online to get information … I mean … everything is transferred online and no one can be asked directly … every place has a robot responding … it has been great that I have not had to use the Internet to connect with the oral health care professional … I have had a chance to ask questions directly from the person … (Parent 6)

#### Personal support

The parents experienced that a personal contact for oral health issues and questions was practical and a great bonus. They expressed that it was convenient that someone contacted them, and they did not need to pay extra effort to seek information by themselves. The parents felt positive that there were personal reminders about the importance of oral health.

It is nice that even one thing can be handled easily because usually the family of a special child has a huge variety of different parties with whom they are in contact … it is great that someone would call me and I don´t need to sort out all the things and try to find contact details … and find out all the things by myself … it is important that someone reminds you of the importance of oral health. Oral health is important but at the same time it is very challenging due to, for example, oral hypersensitivity (Parent 5)

#### Regularity

The parents felt that oral health promotion phone calls were performed at appropriate intervals giving them time to think of questions related with oral health. They also appreciated the long duration of the repeat longitudinal intervention process providing sustainability and support in their daily life.

I thought that phone call would come soon, and I can just wait for it. Phone calls came just in the appropriate intervals so that there was an appropriate quantity of questions to be asked … (Parent 1)

### Individuality of the support

The category “individuality of the support” describes the parental experiences of the received individual support regarding their child’s oral health care. The category included two subcategories: 1) comprehensiveness and 2) essentiality.

#### Comprehensiveness

The parents appreciated the opportunity to contact the dental hygienist counselling person during the whole intervention period. The opportunity to ask questions during the phone calls and the perception that their worries and concerns related with their child’s oral health were addressed was important. Parents also felt that they got answers to all their questions.

I had a chance to ask additional questions every time and it was possible to gain customized answers … it is so that if you have a general material then it is not so personal! (Parent 1)

#### Essentiality

The parents experienced that during the phone calls, their child’s oral health issues were always discussed and that it had been pleasant to gain information and advice that would meet their current situation and needs. They felt that the oral health promotion was designed to address areas of importance and with their current situation considered, and that they were listened to.

There have been occasional challenges in oral health care at home and the situations and challenges have changed over time and child’s age stages … it has been nice to get advice according to the current situation … that has been very nice … (Parent 9)

### Relevancy of the support

The category “relevancy of the support” describes the parents’ experiences of receiving support in various phases of the intervention. The category includes three subcategories which are 1) maintaining awareness, 2) usefulness of the provided information and 3) emphasis on the importance of oral health in everyday life.

#### Maintaining awareness

The parents experienced that the regularly performed phone calls had several advantages. They felt that phone calls were good personal reminders of their child’s oral health situation. The parents expressed that it was important that they got reminders on the importance of oral health in their busy everyday life, thereby making it possible to maintain their child’s oral health care habits.

So … that there has been like regular reminder … that this oral health is an important thing … even though we have a lot of different schedules in our daily life … so it is good and important … (Parent 2)

#### Usefulness of the provided information

The parents experienced that the information they received during the intervention was useful and beneficial for their child’s health. They said that the frequently sent oral health information pamphlet was a convenient repetition regarding oral health issues. They also experienced that the information in this pamphlet was important and useful, and it provided extra information. In contrast, parents also said that the received information was partly repetitious of the already known, basic and self-evident issues concerning oral health. However, they felt that it was still good to repeatedly be provided with this information.

Lot of the received information has been repetitions of already known, however, when you are in the middle of the crisis, which is the situation usually with children with heart disease, it is always a good reminder and repetition of those things that are obvious, because you might forget them, when there are so much going on (Parent 5)

#### Emphasis on the importance of oral health in everyday life

The parents said that the dental hygienist contact strengthened their knowledge in maintaining oral health in children with CHD in everyday life. They also felt that taking care of oral health is an important aspect for all family members. The parents also expressed that without this intervention their knowledge about the importance of oral health care in children with CHD would have been limited.

Taking care of oral health such as toothbrushing has become a basic routine for us. I´m paying much more attention to taking care also of my own oral health … I want everyone in our family to have a good oral health … so that there will be no additional problems because of our actions (Parent 9)

### Other issues raised from the interview material

A question concerning the feedback and development of the intervention was also presented during the interview. The parents felt that the OHPI could also work for other children with systemic disease and that they would recommend other families to participate in the intervention. As mentioned above, the parents appreciated the personal contact. However, it was also proposed that, a social media group as a peer support method could be feasible. Furthermore, parents said that some of the information could be accessible via the Internet in addition to the personal phone calls.

The oral health promotion phone calls lasted between five and twenty minutes. Main parental concerns regarding the child’s oral health during the MI included irregular eating, deficient oral health care routines, difficulties with cooperation during tooth brushing, and child not being invited to a primary health care dental appointment.

## Discussion

The purpose of this study was to explore parents’ perceptions and experiences of an OHPI and use this information to examine the feasibility of the OHPI among parents of children with CHD considered at high-risk for endocarditis.

The current study demonstrates that the parental experience of the OHPI was positive overall and that the OHPI was feasible from a parental perspective. Many advantages from the participation in the OHPI were mentioned. Parents experienced the oral health promotion with frequent phone calls to be effortless and easy to follow. They also recommended the intervention to other parents. This result is in line with another study that investigated parents’ perception on received health promotion (Woo Baidal et al., 2013). In Finland, all paediatric heart surgery is performed in the Children´s Hospital of the Helsinki University Hospital. Consequently, the most feasible timing of recruitment and baseline oral health promotion was to perform this in conjunction with the heart procedure, and to conduct the following frequent oral health promotions by telephone for geographical reasons.

Children with complex heart conditions commonly undergo prolonged in-hospital treatment periods during the neonatal stage that may be challenged by readmissions to intensive care and further prolongation of in-hospital care. The interviewed parents expressed feelings of stress, as being in the middle of a crisis. Parental recollections from the first contact of study recruitment and baseline OHPI were blurry. The perioperative paediatric intensive and cardiac ward care was substantial and similar for the parents participating in this qualitative study compared with the ORALPEDHEART-study population. The findings from our study are in line with previous studies reporting parental mixed emotions between the time of CHD diagnosis and discharge from hospital following heart surgery (Sook et al., 2018). Parents also report a wide range of emotions including fear during child intensive care (Dahav and Sjöström-Strand, 2018; Cantwell-Bartl & Tibballs, 2013). Our study results are relevant when planning the timing of a first contact for an intervention targeting parents of children with a severe systemic health condition. In the future, the first contact with oral health promotion personnel might be more suitable to conduct after hospital discharge. However, our study also indicated that some parents emphasized that it was pleasant to receive more general and less dramatic health information and support at the early stage.

Information regarding health promotion is available on the Internet and individuals are encouraged to access this information by themselves. The Internet does not, however, provide the opportunity for personalized counselling that can be provided during phone call discussions. The results from this study indicate that parents value individual oral support offered by a professional. The study also suggest that parents experienced repeat telephone contacts during the 18-month period as a convenient way to update relevant information that they probably would not access otherwise. The parents experienced that the repeat counselling supported them to maintain oral health routines in the middle of a hectic everyday life. Although the information they received was mostly repetition, it was experienced as an important reminder.

The information collected from the phone calls indicated that the parents’ concerns regarding child oral health was general and without specific concerns expressed specifically relating oral health with context of child CHD and long-term risk of endocarditis. Parental needs of specialized oral health information for child CHD seems, thus, unmet in standard CHD care. Repeat counselling during the early 2-years of life helps these families optimize oral health practices into everyday life routines with a potential for long-term benefits.

When assessing the validity of a qualitative study, the focus is on its trustworthiness, which in relation to inductive content analysis needs to be considered during the data collection, analysis, and presentation of the results (Elo et al., 2014). Phone call interviews were chosen as a data collection method because the families that participated in this study live across Finland, and it would have been difficult to organize face-to-face interviews. Moreover, it was evaluated that by phone calls it was possible to gain the information on parental experiences similar to face-to-face interviews. However, performing the interviews face-to-face might have provided more insight into the parents’ nonverbal behaviour. In the future, virtual personal face-to-face counselling sessions might provide benefits related with this.

One of the things that increases the trustworthiness and especially credibility of this study is that an independent external oral health care professional not involved with CHD children oral health performed the phone call interviews precluding the possibility of bias caused by the person conducting the oral health counselling intervention (Korstjens & Moser, 2018). In the MI technique, parents moderate the discussion, which may include other child health concerns that may impact on the content of the oral health promotion intervention. However, the oral health promotion sessions followed a standardized written information protocol on oral health of children with CHD.

In a qualitative study, the sample must be appropriate and representative and have knowledge of the topic of interest (Elo et al., 2014). Participating parents were purposefully selected based on knowledge and experience about the OHPI. The representativeness of the interviewed parents in relation to parents participating in the ORALPEDHEART-study overall adds adequacy to this qualitative study. However, this study may still include selection bias as participating parents might show a more positive attitude towards the intervention than non-participating parents might.

It has been suggested, that in inductive analysis one researcher should take the responsibility over analysis, while other researchers follow the process and outcomes (Elo et al., 2014). To strengthen the credibility of the study, interview data was analysed from specific to general by three researchers representing different professional fields. Researchers continuously discussed the classification of the sub- and main categories. However, the first author was familiar with the interviewed participants, which has to be considered when evaluating the confirmability of the results. To make the analysing process transferable, an example of analysing process of one main category is presented. Moreover, quotations are presented in the result section to indicate the trustworthiness of the results and to show a connection between the data, results, and conclusions.

The collected qualitative data allowed in depth assessments of the parents’ experiences and perceptions of the intervention, which the authors consider a strength of this study. This provided the opportunity to assess feasibility of the developed intervention from the perspective of the participating parent.

In conclusion, this is to our knowledge the first study exploring the perceptions and experiences of parents of children with CHD participating in an OHPI. In all, parents were satisfied with the intervention with no major modifications needed. The study shows that parents experience the OHPI to be important and feasible to implement in everyday life in children with CHD. However, in the future, the timing of the first contact and applying additional web-based support systems should be evaluated. Our findings may be of interest to other health care professional who are developing health promotion interventions for children with systemic diseases.
